# Coronary flow capacity and survival prediction after revascularization: physiological basis and clinical implications

**DOI:** 10.1093/eurheartj/ehad579

**Published:** 2023-08-27

**Authors:** K Lance Gould, Nils P Johnson, Amanda E Roby, Linh Bui, Danai Kitkungvan, Monica B Patel, Tung Nguyen, Richard Kirkeeide, Mary Haynie, Salman A Arain, Konstantinos Charitakis, Abhijeet Dhoble, Richard Smalling, Angelo Nascimbene, Marwan Jumean, Sachin Kumar, Biswajit Kar, Stefano Sdringola, Anthony Estrera, Igor Gregoric, Dejian Lai, Ruosha Li, David McPherson, Jagat Narula

**Affiliations:** Department of Medicine, Division of Cardiology, Weatherhead P.E.T. Center, McGovern Medical School, University of Texas Health Science Center, and Memorial Hermann Hospital, 6431 Fannin St., Room MSB 4.256, Houston, TX 77030, USA; Department of Medicine, Division of Cardiology, McGovern Medical School, University of Texas Health Science Center, and Memorial Hermann Hospital, 6431 Fannin St, Rm 4.256 MSB, Houston, TX 77005, USA; Department of Medicine, Division of Cardiology, Weatherhead P.E.T. Center, McGovern Medical School, University of Texas Health Science Center, and Memorial Hermann Hospital, 6431 Fannin St., Room MSB 4.256, Houston, TX 77030, USA; Department of Medicine, Division of Cardiology, McGovern Medical School, University of Texas Health Science Center, and Memorial Hermann Hospital, 6431 Fannin St, Rm 4.256 MSB, Houston, TX 77005, USA; Department of Medicine, Division of Cardiology, Weatherhead P.E.T. Center, McGovern Medical School, University of Texas Health Science Center, and Memorial Hermann Hospital, 6431 Fannin St., Room MSB 4.256, Houston, TX 77030, USA; Department of Medicine, Division of Cardiology, McGovern Medical School, University of Texas Health Science Center, and Memorial Hermann Hospital, 6431 Fannin St, Rm 4.256 MSB, Houston, TX 77005, USA; Department of Medicine, Division of Cardiology, Weatherhead P.E.T. Center, McGovern Medical School, University of Texas Health Science Center, and Memorial Hermann Hospital, 6431 Fannin St., Room MSB 4.256, Houston, TX 77030, USA; Department of Medicine, Division of Cardiology, McGovern Medical School, University of Texas Health Science Center, and Memorial Hermann Hospital, 6431 Fannin St, Rm 4.256 MSB, Houston, TX 77005, USA; Department of Medicine, Division of Cardiology, Weatherhead P.E.T. Center, McGovern Medical School, University of Texas Health Science Center, and Memorial Hermann Hospital, 6431 Fannin St., Room MSB 4.256, Houston, TX 77030, USA; Department of Medicine, Division of Cardiology, McGovern Medical School, University of Texas Health Science Center, and Memorial Hermann Hospital, 6431 Fannin St, Rm 4.256 MSB, Houston, TX 77005, USA; Department of Medicine, Division of Cardiology, Weatherhead P.E.T. Center, McGovern Medical School, University of Texas Health Science Center, and Memorial Hermann Hospital, 6431 Fannin St., Room MSB 4.256, Houston, TX 77030, USA; Department of Medicine, Division of Cardiology, McGovern Medical School, University of Texas Health Science Center, and Memorial Hermann Hospital, 6431 Fannin St, Rm 4.256 MSB, Houston, TX 77005, USA; Department of Medicine, Division of Cardiology, Weatherhead P.E.T. Center, McGovern Medical School, University of Texas Health Science Center, and Memorial Hermann Hospital, 6431 Fannin St., Room MSB 4.256, Houston, TX 77030, USA; Department of Medicine, Division of Cardiology, McGovern Medical School, University of Texas Health Science Center, and Memorial Hermann Hospital, 6431 Fannin St, Rm 4.256 MSB, Houston, TX 77005, USA; Department of Medicine, Division of Cardiology, Weatherhead P.E.T. Center, McGovern Medical School, University of Texas Health Science Center, and Memorial Hermann Hospital, 6431 Fannin St., Room MSB 4.256, Houston, TX 77030, USA; Department of Medicine, Division of Cardiology, McGovern Medical School, University of Texas Health Science Center, and Memorial Hermann Hospital, 6431 Fannin St, Rm 4.256 MSB, Houston, TX 77005, USA; Department of Medicine, Division of Cardiology, Weatherhead P.E.T. Center, McGovern Medical School, University of Texas Health Science Center, and Memorial Hermann Hospital, 6431 Fannin St., Room MSB 4.256, Houston, TX 77030, USA; Department of Medicine, Division of Cardiology, McGovern Medical School, University of Texas Health Science Center, and Memorial Hermann Hospital, 6431 Fannin St, Rm 4.256 MSB, Houston, TX 77005, USA; Department of Medicine, Division of Cardiology, McGovern Medical School, University of Texas Health Science Center, and Memorial Hermann Hospital, 6431 Fannin St, Rm 4.256 MSB, Houston, TX 77005, USA; Department of Medicine, Division of Cardiology, McGovern Medical School, University of Texas Health Science Center, and Memorial Hermann Hospital, 6431 Fannin St, Rm 4.256 MSB, Houston, TX 77005, USA; Department of Medicine, Division of Cardiology, McGovern Medical School, University of Texas Health Science Center, and Memorial Hermann Hospital, 6431 Fannin St, Rm 4.256 MSB, Houston, TX 77005, USA; Department of Medicine, Division of Cardiology, McGovern Medical School, University of Texas Health Science Center, and Memorial Hermann Hospital, 6431 Fannin St, Rm 4.256 MSB, Houston, TX 77005, USA; Department of Advanced Cardiopulmonary Therapies and Transplantation, McGovern Medical School, University of Texas Health Science Center, and Memorial Hermann Hospital, Houston, TX, USA; Department of Advanced Cardiopulmonary Therapies and Transplantation, McGovern Medical School, University of Texas Health Science Center, and Memorial Hermann Hospital, Houston, TX, USA; Department of Advanced Cardiopulmonary Therapies and Transplantation, McGovern Medical School, University of Texas Health Science Center, and Memorial Hermann Hospital, Houston, TX, USA; Department of Advanced Cardiopulmonary Therapies and Transplantation, McGovern Medical School, University of Texas Health Science Center, and Memorial Hermann Hospital, Houston, TX, USA; Department of Medicine, Division of Cardiology, McGovern Medical School, University of Texas Health Science Center, and Memorial Hermann Hospital, 6431 Fannin St, Rm 4.256 MSB, Houston, TX 77005, USA; Department of Cardiothoracic Vascular Surgery, McGovern Medical School, University of Texas Health Science Center, Memorial Hermann Hospital, Houston, TX, USA; Department of Advanced Cardiopulmonary Therapies and Transplantation, McGovern Medical School, University of Texas Health Science Center, and Memorial Hermann Hospital, Houston, TX, USA; Department of Biostatistics and Data Science, University of Texas School of Public Health—Houston, Houston, TX, USA; Department of Biostatistics and Data Science, University of Texas School of Public Health—Houston, Houston, TX, USA; Department of Medicine, Division of Cardiology, McGovern Medical School, University of Texas Health Science Center, and Memorial Hermann Hospital, 6431 Fannin St, Rm 4.256 MSB, Houston, TX 77005, USA; Department of Medicine, Division of Cardiology, Weatherhead P.E.T. Center, McGovern Medical School, University of Texas Health Science Center, and Memorial Hermann Hospital, 6431 Fannin St., Room MSB 4.256, Houston, TX 77030, USA; Department of Medicine, Division of Cardiology, McGovern Medical School, University of Texas Health Science Center, and Memorial Hermann Hospital, 6431 Fannin St, Rm 4.256 MSB, Houston, TX 77005, USA

**Keywords:** Coronary artery disease, Coronary flow reserve, PET imaging, Myocardial perfusion, Myocardial ischaemia, Coronary bypass surgery

## Abstract

**Background and Aims:**

Coronary flow capacity (CFC) is associated with an observed 10-year survival probability for individual patients before and after actual revascularization for comparison to virtual hypothetical ideal complete revascularization.

**Methods:**

Stress myocardial perfusion (mL/min/g) and coronary flow reserve (CFR) per pixel were quantified in 6979 coronary artery disease (CAD) subjects using Rb-82 positron emission tomography (PET) for CFC maps of artery-specific size-severity abnormalities expressed as percent left ventricle with prospective follow-up to define survival probability per-decade as fraction of 1.0.

**Results:**

Severely reduced CFC in 6979 subjects predicted low survival probability that improved by 42% after revascularization compared with no revascularization for comparable severity (*P* = .0015). For 283 pre-and-post-procedure PET pairs, severely reduced regional CFC-associated survival probability improved heterogeneously after revascularization (*P* < .001), more so after bypass surgery than percutaneous coronary interventions (*P* < .001) but normalized in only 5.7%; non-severe baseline CFC or survival probability did not improve compared with severe CFC (*P* = .00001). Observed CFC-associated survival probability after actual revascularization was lower than virtual ideal hypothetical complete post-revascularization survival probability due to residual CAD or failed revascularization (*P* < .001) unrelated to gender or microvascular dysfunction. Severely reduced CFC in 2552 post-revascularization subjects associated with low survival probability also improved after repeat revascularization compared with no repeat procedures (*P* = .025).

**Conclusions:**

Severely reduced CFC and associated observed survival probability improved after first and repeat revascularization compared with no revascularization for comparable CFC severity. Non-severe CFC showed no benefit. Discordance between observed actual and virtual hypothetical post-revascularization survival probability revealed residual CAD or failed revascularization.

## Structured Graphical Abstract

**Structured Graphical Abstract:**
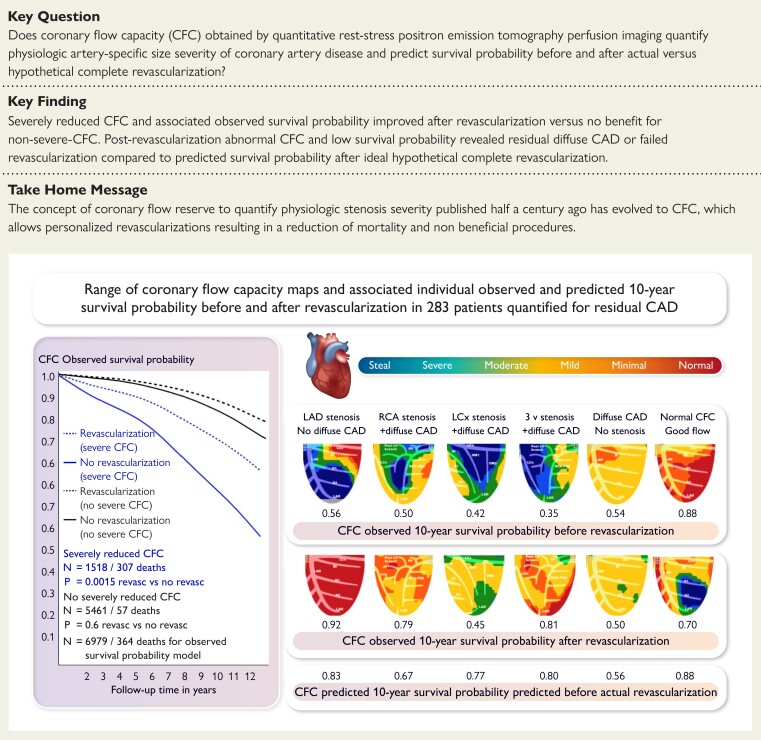
Coronary flow capacity (CFC) and survival prediction after revascularization: physiological basis and clinical implications. CFC maps in one view before (upper row) and after revascularization (lower row) show residual diffuse coronary artery disease (CAD), stenosis, and incomplete or inappropriate revascularization as examples from 283 pre-post-revascularization positron emission tomography (PET) pairs. The 10-year survival probability is determined as a fraction of one for normal CFC (all red) by the proportional distribution of regional size-severity CFC abnormalities. The individual observed survival probabilities for each CFC map before and after actual coronary revascularization are listed below each CFC map. The virtual survival probability predicted for the baseline CFC map after virtual theoretical ideal complete revascularization is listed in the lowest (3rd) row for each case. The plots show the observed survival probability of 6979 PET cases with and without severely reduced CFC with and without non-randomized revascularization followed over 12 years as the database from which individual survival probability is determined by multi-variable CFC components by Cox regression modelling. LAD, left anterior descending coronary artery; LCx, left circumflex artery; RCA, right coronary artery.

**See the editorial comment for this article ‘Leveraging global coronary flow assessments to inform revascularization benefit in chronic coronary disease: time to test total heart flow’, by V.R. Taqueti, https://doi.org/10.1093/eurheartj/ehad812.**

## Introduction

While coronary revascularization is intended to improve myocardial perfusion and reduce ischaemia, randomized revascularization trials have not demonstrated improved survival compared with medical treatment.^1–3^ The artery-specific map of coronary flow capacity (CFC) by quantitative rest-stress positron emission tomography (PET) perfusion imaging associates with or predicts survival probability in chronic coronary artery disease (CAD).^4–12^ Severely reduced CFC and associated low survival probability are significantly improved after revascularization compared to no revascularization for comparable severity in large non-randomized cohorts.^4–6^

Conversely, non-severe CFC regions associated with low mortality risk show no improvement after revascularization.^9,10^ Similarly, severe perfusion abnormalities with no improvement after revascularization have no improved survival.^4–6,10–12^ In patients with severely reduced CFC and low survival probability that improves after revascularization, substantial unquantified residual CAD commonly remains with abnormal CFC and associated residual limited survival probability.^4–6,10–12^ The quantitative burden of residual focal or diffuse CAD, incomplete or failed revascularization, and associated survival probability before and after revascularization remain undefined and unpredictable in individuals.

Based on these previously reported observations, we tested the following hypothesis. Severely reduced CFC associates with or predicts low 10-year survival probability that is significantly improved after revascularization compared to no revascularization for comparable CFC severity. However, improvement may be heterogeneous or limited by residual diffuse or focal CAD that has not been previously quantified or virtually predicted before revascularization. The analysis requires three steps.

First, in a cohort of 6979 routine diagnostic quantitative rest-stress PET cases followed over 12 years, extended data indicate that CFC maps of the size-severity range of stress perfusion in mL/min/g and coronary flow reserve (CFR) per pixel as % of left ventricle (LV) associate with or predict survival probability. As an extension of this observation, we hypothesized that low survival probability associated with severely reduced CFC might be substantially modified by the size-severity of surrounding normal or mildly reduced CFC regions reflecting diffuse disease or multiple mild to moderate stenosis in a continuum of relatively limited survival probability. Consequently, revascularization might improve survival probability to a greater or lesser extent depending on the range of residual CFC size-severity abnormalities reflecting residual focal or diffuse CAD to explain variable or limited revascularization effects on mortality. As a specific test of this hypothesis in individual patients, we analysed 283 pre- and post-revascularization PET pairs of CFC maps and associated survival probability for their pre- to post-revascularization changes observed for variable effects of revascularization in individual patients.

Second, we hypothesized that *Virtual* survival probability can be predicted for ideal, hypothetical, complete revascularization by replacing the % of severely reduced CFC pixels in the pre-revascularization CFC map with normal CFC severity pixels in the Cox regression modelling of survival probability. This CFC-derived *Virtual* survival probability after hypothetical, ideal, complete revascularization can then be compared with CFC-associated *Observed* survival probability after actual revascularization. Discordance between the CFC-associated *Observed* survival probability after actual revascularization and the CFC *Virtually* predicted post-revascularization survival probability would indicate suboptimal revascularization due to residual diffuse CAD, incomplete or failed revascularization, or progressive CAD. The *Virtual* survival probability for the pre-revascularization CFC would therefore suggest the optimal potential effect of revascularization on survival probability in an individual patient before doing the procedure.

Finally, in 2552 post-revascularization PET patients, we also hypothesized that CFC maps in patients with prior revascularization may have residual severe regional abnormalities associated with poor survival probability that improve after repeat revascularization. However, substantial residual limited survival probability may remain due to more residual CAD than patients without prior revascularization.

## Methods

At the Weatherhead PET Center, McGovern Medical School, University of Texas (UT) Health Science Center, Houston, 6979 routine diagnostic rest-stress, quantitative, myocardial perfusion PETs were done on consecutive patients after informed consent in prospective ongoing research with systematic follow-up over 12 years by trained, blinded research assistants as approved by our institutional Committee for Protection of Human Subjects (CPHS). Complete medical history is entered into a dedicated Health Insurance Portability and Accountability Act of 1996 (HIPAA)-compliant relational database.^4–6,13^ For every PET, a blood sample is tested for caffeine. Systematic repeat PET after revascularization is done under a UT CPHS-approved protocol with written consent and funded by the UT Weatherhead Endowment.

### Cardiac positron emission tomography acquisition and analysis

All patients are instructed to fast for 4 h and abstain from caffeine and cigarettes for 24 h. Cardiac PET-computed tomography (CT) was acquired as previously reported (Discovery ST 16-slice GE Healthcare PET-CT scanner in 2-dimensional mode, Waukesha, Wisconsin, or a United Imaging solid state 3-D PET-CT, Houston, TX) after intravenous 30–50 mCi (1110–1850 MBq) of Rb-82 (Bracco Diagnostics, Princeton, New Jersey).^4–6,13^ In 196 paired PETs acquired in the same patient on same day, rest and stress myocardial perfusion in mL/min/g on these different scanners are equivalent for Rb-82 (R^2^ = 0.99, coefficient of variance ±12%). Attenuation correction used cine CT tube current modulation with reduced radiation dose, co-registration, and validated region-of-interest for arterial input.^4–6,13^ Only 0.7% failed to yield quantitative perfusion data due to equipment failure or subclavian vein occlusion precluding arterial input. Standard pharmacological stress used dipyridamole infusion (0.142 mg/kg/min) over 4 min with 4-min-wait to peak vasodilation for second infusion of Rb-82.^4–6,13^

**Figure 1:**
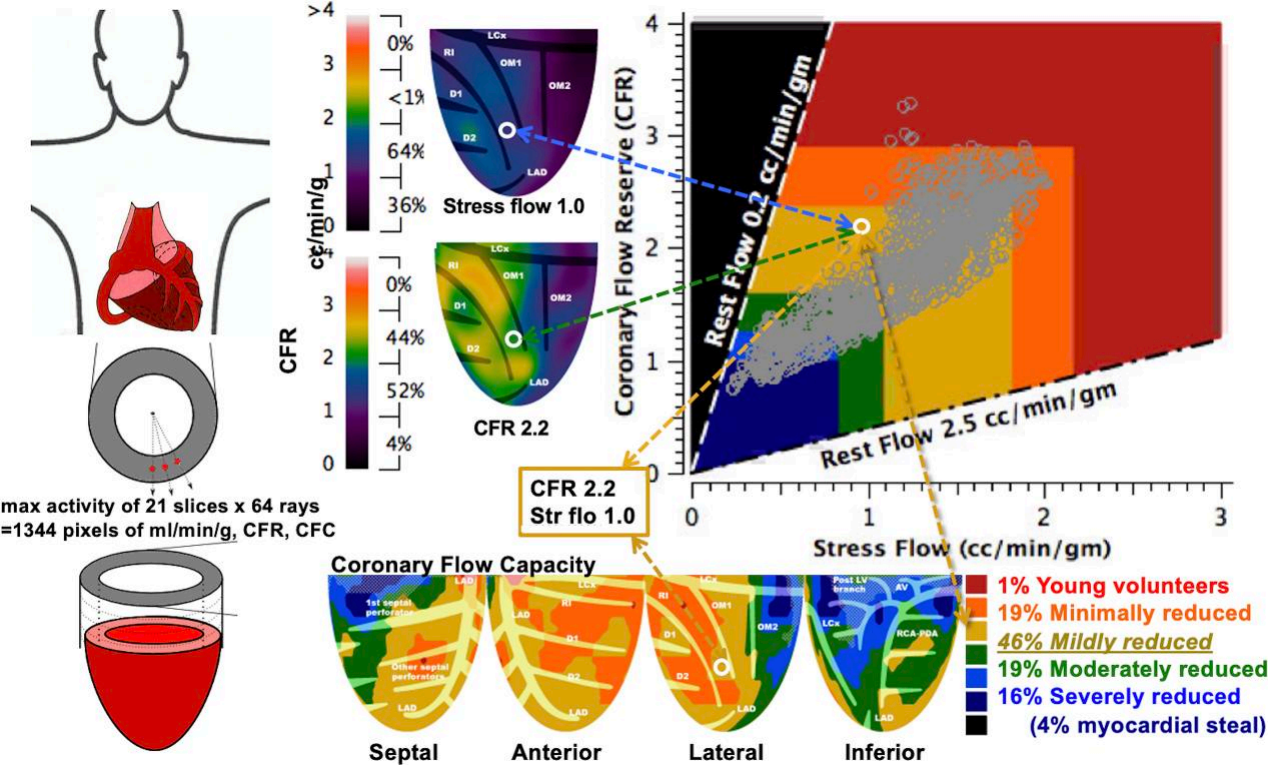
Regional pixel values of coronary flow reserve and stress (cc/min/g) are colour coded in prespecified ranges and mapped into their left ventricle location to produce a coronary flow capacity map for % of the left ventricle as excellent (red), adequate (orange), mildly reduced (yellow), moderately reduced (green), severely reduced (blue), or myocardial steal (dark blue) in artery specific distributions down to tertiary branches as further defined in the text

The regional CFC map (*Figure 1*) of the LV combines regional pixel values of CFR and stress (mL/min/g).^4–6,13^ The wide range of stress myocardial perfusion in mL/min/g and CFR values for each of 1344 radial pixels comprise vast numbers of possible stress perfusion and CFR pixel combinations that are compressed into the following objectively determined ranges of combined values for each regional pixel by ROC analysis for specific clinical groups as reported^4–6,13^ summarized as follows:

*Excellent*, coloured red from healthy young volunteers (CFR >2.9 and stress perfusion >2.17 mL/min/g).

*Adequate*, coloured orange from asymptomatic patients with risk factors without known CAD (CFR >2.38 to 2.9 and stress perfusion >1.82 to 2.17).

*Mildly reduced*, yellow from patients with known CAD without symptoms of ischaemia (CFR >1.6 to 2.38 and stress perfusion >1.09 to 1.82).

*Moderately reduced*, coloured green from patients with either a regional stress defect or angina or ST depression ≥ 1 mm during dipyridamole stress (CFR >1.27 to 1.6 and stress perfusion >0.83 to 1.09).

*Severely reduced*, coloured blue from patients with two of these three manifestations of ischaemia (CFR 1.0 to 1.27 and stress perfusion ≤0.83). *Myocardial steal*, coloured dark blue (defined as CFR <1.0).^4–6,13^

*Myocardial scar,* coloured as grey hatch marks, defined as fixed resting and stress perfusion ≤0.3 mL/min/g as % of LV.

Each colour-coded pixel is spatially mapped back onto its LV location with per cent of LV calculated for each range of combined both CFR and stress perfusion pixel values listed in the CFC colour histogram bar. The regional, colour-coded 1344 pixels provide integrated, size-severity quantification for each specific coronary artery distribution down to tertiary branches using Food and Drug Administration (FDA)-approved software (FDA K202679).^4–6,13^ The coefficient of variance for mL/min/g is ±10% on serial rest–rest and stress–stress images in the same patient minutes apart and <±1% on the Kolmogorov–Smirnov (KS) test of serial CFC histograms in the same patient.^13^

### *Observed* survival probability associated with 6979 coronary flow capacity maps by positron emission tomography

As previously reported, the *Observed* 10-year survival probability associated with individual PET CFC maps was determined as a fraction of 1.0 by multi-variable Cox regression modelling of 6979 rest-stress PET with >90-day follow-up for time to all-cause mortality in the prospective database with follow-up over 12 years.^6^ Covariates in the Cox model were the combined CFC size-severity as % of LV with CFCmild, CFCsevere (i.e. moderate and/or severe), and myocardial scar, using CFCnormal (i.e. normal and/or minimal) as the reference in the Cox model.^6^

### *Virtual* survival probability after hypothetical, ideal, complete revascularization

The *Virtual* survival probability after optimal, ideal, complete, hypothetical revascularization was calculated in the Cox regression model by replacing the % of severely reduced CFC pixels in pre-revascularization CFC maps with % of LV as normal CFC pixels surrounding the severe CFC abnormality. Therefore, the *Optimal Virtual* survival probability reflects the likelihood of improved survival probability after ideal complete revascularization compared to the *Observed* CFC-associated survival probability after actual revascularization to aid decision-making on revascularization before procedures were done. For frequent post-revascularization residual CAD or incomplete revascularization, a *Realistic Virtual survival probability* was determined in the Cox model by replacing % LV with severely reduced CFC in the baseline pre-revascularization PET by the proportionate ratio of regional distribution of CFC mild pixels to normal CFC pixels outside the CFC severely reduced pixels while keeping constant the % of LV with mildly reduced CFC and scar.

The *Optimal* and *Realistic Virtual* survival probabilities bracketed the range of expected post-revascularization survival probabilities. Discordances between the *Observed* CFC-associated survival probability after actual revascularization and the *Virtual* survival probabilities indicate risk of residual diffuse narrowing, stenosis, or incomplete revascularization.

### Clinical follow-up

A programmed, prospective follow-up consent form approved by our Committee for Protection of Human Patients was obtained after every PET. Blinded research assistants systematically and continuously record clinical events from clinic or hospital records, mailed questionnaires, phone calls, email, or web searches of newspaper obituaries as an ongoing monthly routine process, repeated three times for initial non-responders.^4–6,13^ A team of cardiologists, experienced research nurses, and experienced research assistants blinded to PET data adjudicated outcomes. All-cause death was used as a definitive, hard outcome to avoid the well-recognized bias in determining cause of death or controversy in defining myocardial infarction (MI).

### Analysis schema for coronary flow capacity severity and survival probability (*Table 1*)

Data analyses were performed in the following steps to address study objectives outlined in the introduction.

**Table 1:** Analysis schema

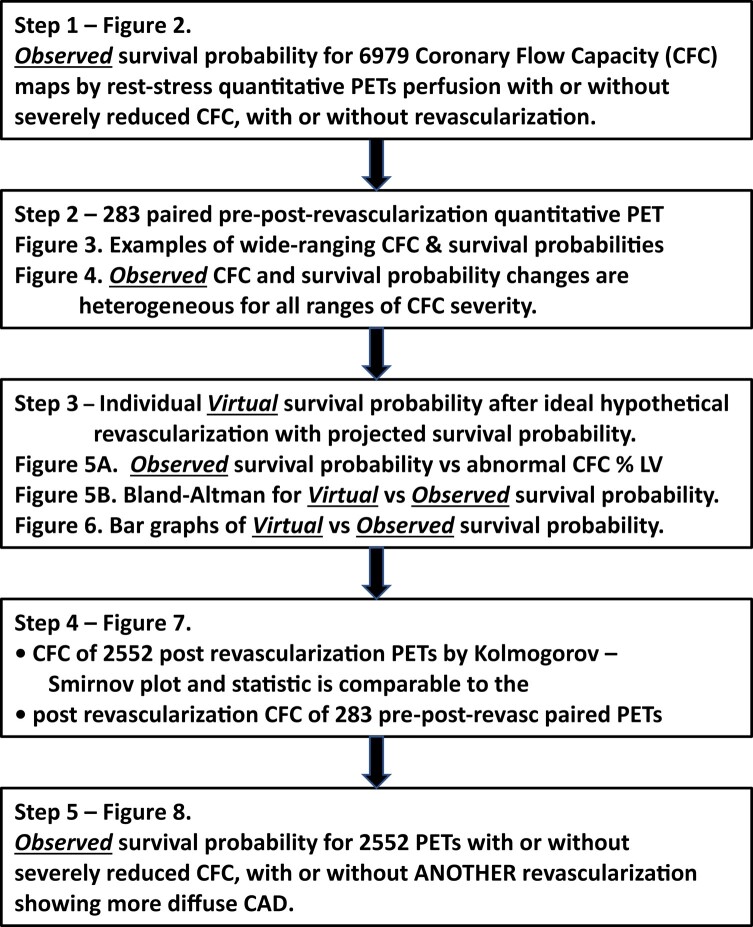

**Figure 2:**
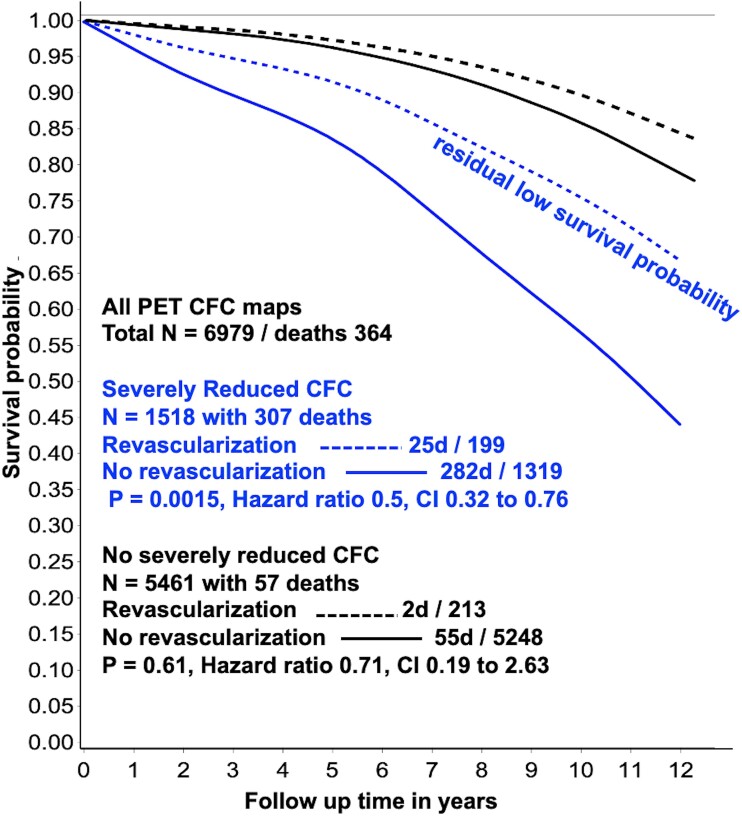
Survival probability for severely reduced (blue lines) and non-severely reduced coronary flow reserve (black lines) with (dashed lines) and without (solid lines) revascularization by multi-variable Cox regression analysis of 6979 coronary flow reserve maps over 12 years follow-up. While survival probability is significantly improved after revascularization for severely reduced coronary flow reserve, residual reduced survival probability persists. CI, confidence intervals

**Step 1**. Multi-variable Cox regression modelling for *Observed* survival probability as a fraction of 1.0 over 12 years associated with 6979 CFC maps of routine rest-stress diagnostic quantitative PET with and without severely reduced CFC, with and without revascularization as previously reported.^6^ (*Figure 2*).

**Figure 3:**
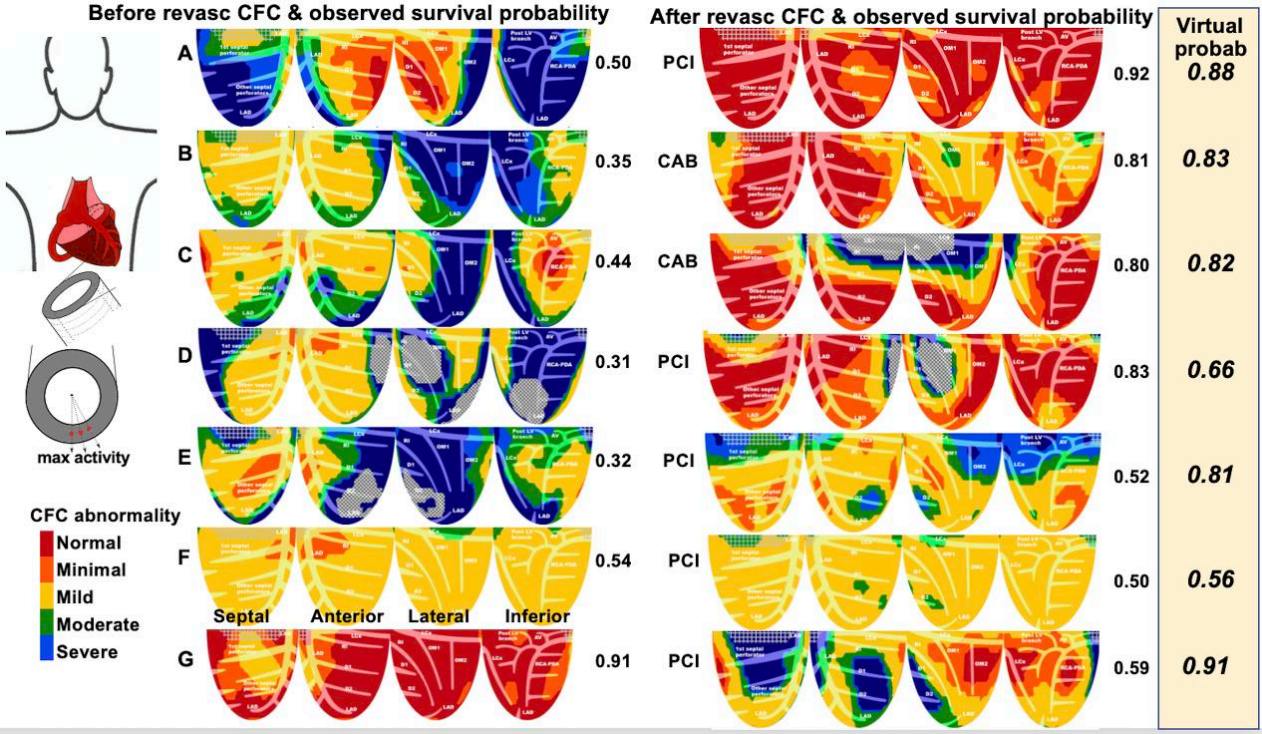
Coronary flow capacity maps show the range of quantitative regional severity-size of perfusion abnormalities in a set of 283 cases with coronary flow capacity maps before and after revascularization with coronary flow capacity derived individual *Observed* coronary flow capacity associated survival probability after actual revascularization beside each image. The *Optimal Virtual* survival probability after optimal, ideal, complete hypothetical revascularization for each individual is listed in italics of the beige column

**Figure 4:**
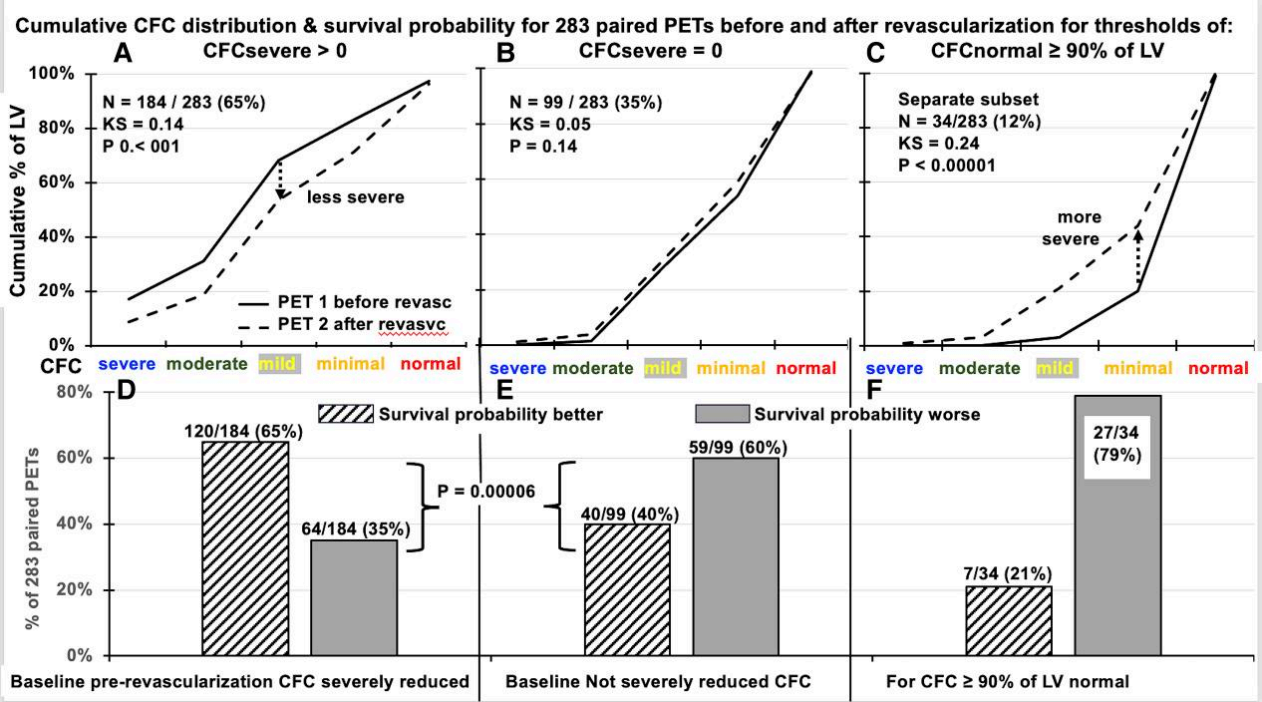
Comparisons of coronary flow reserve size-severity cumulative histograms of PET before and after revascularization by the Kolmogorov–Smirnov test for the 283 pre-post-revascularization paired PET’s, and pre-revascularization PET 1 having (*A*) severely reduced coronary flow reserve; (*B*) no severely reduced coronary flow reserve in the pre-revascularization PET 1; and normal CFC for ≥90% of left ventricle in the pre-revascularization PET 1 (*C*), showing improved (*D*) or worsened survival probability for baseline severely reduced coronary flow reserve (*E*), and markedly worse survival probability after revascularization of no severely reduced coronary flow reserve (*F*)

**Step 2.** The CFC maps were compared from 283 pre- and post-revascularization CFC pairs. The range of changes in CFC maps is displayed in colour (*Figure 3*) and CFC severity histogram plots were systematically compared using KS tests (*Figure 4*). The *Observed* CFC-associated survival probability as a fraction of 1.0 for each pre- and post-revascularization CFC pair was better or worse after revascularization as a % of patients having pre-revascularization CFC that was severely reduced, not severely reduced, or normal for ≥90% of LV (*Figure 4*).

**Figure 5:**
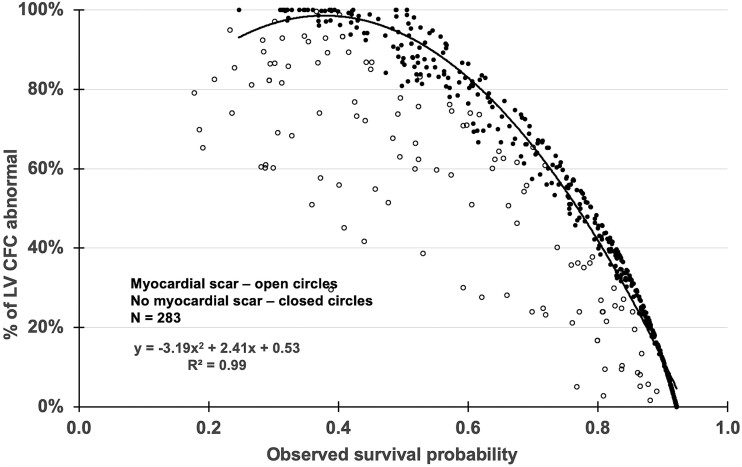
Observed survival probability associated with abnormal coronary flow reserve as % of left ventricle. For 283 paired PET before and after revascularization in the same patient, the observed survival probability is lower (worse) with increasing size of abnormal coronary flow reserve defined as mild, moderate, or severe as % of left ventricle, that is worse with myocardial scar

**Figure 6:**
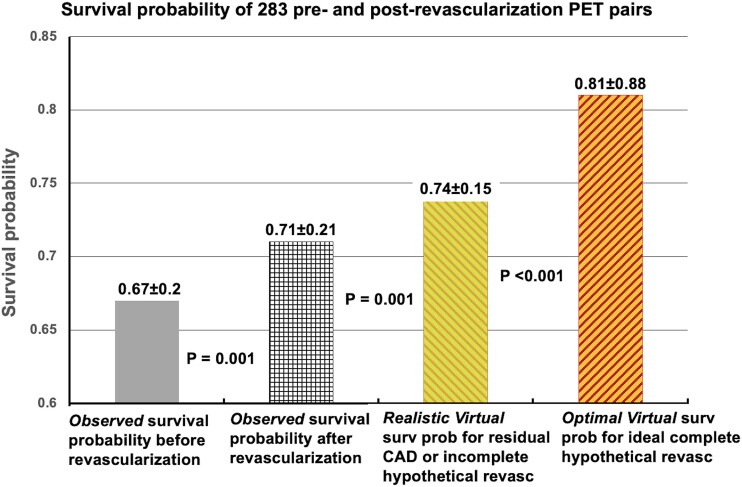
*Virtual* survival probabilities after hypothetical revascularization vs. *Observed* survival probability after actual revascularization

**Step 3**. The *Observed* survival probabilities before and after revascularization of the 283 PET pairs were compared to % of LV with abnormal CFC or % myocardial scar (*Figure 5*). *Observed* and *Virtual* survival probability were compared to assess the residual risk from residual diffuse CAD or incomplete revascularization that limits improvement of CFC or associated survival probability (*Figure 6*).

**Figure 7:**
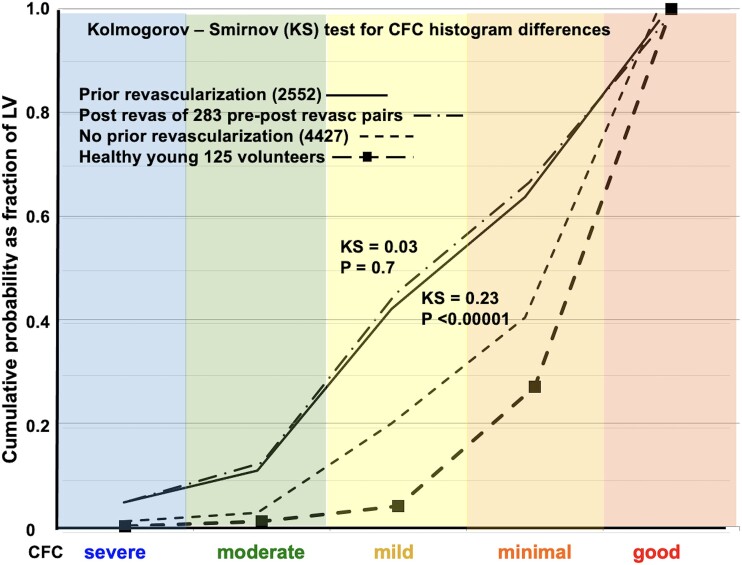
Kolmogorov—Smirnov statistic for cumulative histogram distribution of coronary flow capacity severity 2552 cases with revascularization before PET (solid line) vs. 4427 without revascularization before PET (fine dashed line) vs. the post revascularization PET of the 283 pre- post-revascularization PET pairs (dash-dot line) vs. healthy young volunteers without risk factors (heavy dashed line). Positron emission tomography after prior revascularization has substantial residual diffuse (mild) and focal (moderate or severe) coronary flow capacity abnormalities that are significantly more severe than PET with no prior revascularization or healthy volunteers

**Step 4**. Comparison of CFC severity histograms by KS test was undertaken for the following patient categories: Healthy young volunteers, patients with or at risk of CAD with no prior revascularization, patients with prior revascularization, the 283 pre-revascularization CFC maps of pre-post-revascularization CFC pairs to document comparable CFC severity of the latter two groups. (*Figure 7*)

**Figure 8:**
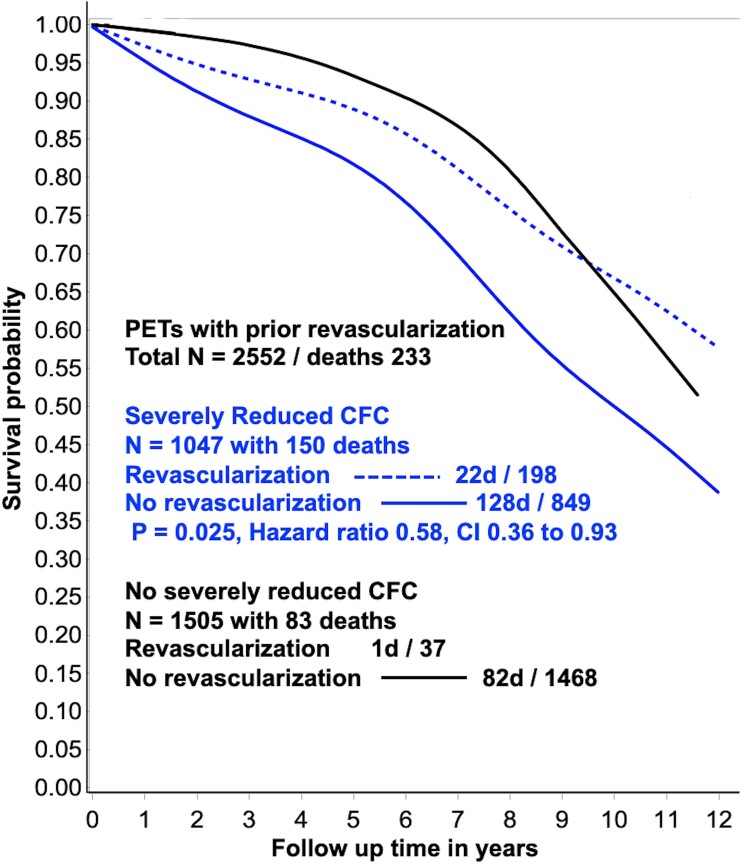
Multi-variable Cox regression analysis of 2552 coronary flow reserve maps after prior revascularization with 12 years follow-up for, *Observed* coronary flow reserve associated survival probability for severely reduced (blue lines) and non-severely reduced coronary flow reserve (black lines) with (dashed lines) and without (solid lines) revascularization. While survival probability is significantly improved for severely reduced coronary flow reserve after another revascularization, substantially reduced survival probability persists (dashed blue line) that is comparable to the post-revascularization survival probability with no severely reduced coronary flow reserve (solid black lines) after the first revascularization. The numbers with no severely reduced coronary flow reserve after the first revascularization having another revascularization are too small for statistical analysis. CI, confidence intervals

**Step 5.** Multi-variable Cox regression modelling for observed survival probability over 12 years derived from 2552 CFC maps of patients with prior revascularization with and without severely reduced CFC, with and without repeat revascularization (*Figure 8*).

### Statistical analysis

For continuous variables, two-tailed tests with *P* < .05 were considered statistically significant for rejecting the null hypothesis. Chi-square was used for a significance of discrete variables. Unpaired *t*-tests with unequal variance between groups were used for continuous variables. As previously reported,^3–6,13,14^ SAS 9.4 was used for multiple variable Cox regression modelling of both severely reduced CFC and non-severe CFC with time-dependent covariates of revascularization after PET and time to all-cause-death after PET on per patient basis with and without post-PET percutaneous coronary intervention (PCI) or coronary artery bypass graft (CABG) surgery including age and gender covariates. KS tests compared histogram distributions between groups in colour-coded ranges of relative regional CFC distribution as % of LV as previously reported.^13,14^

## Results

As a tertiary care academic centre with advanced quantitative perfusion imaging and complex revascularization services, our large referral cohort of 6979 routine, diagnostic, rest-stress quantitative PET perfusion images included high prevalence of known (40%), or suspected CAD, high-risk factor burden or coronary calcium (77%) scores as previously reported.^3–6,13^ Over 60% are quantitatively not-normal, but only 21% had severely reduced CFC and only 12% were revascularized depending on clinical judgement, comorbidities, size, and proximal vs. distal stress defects. Severely reduced CFC was associated with low survival probability (*Figure 2* solid blue line) that was significantly improved after revascularization (*Figure 2* dashed blue line) (*P* = .0015) over 12 years compared to no revascularization for comparable severity (*Figure 2*); average mortality was 21.4% without vs. 12.5% with revascularization for comparable pre-procedure CFC severity, a reduction of 42%. While improved after revascularization, the survival probability remained substantially reduced (*Figure 2* dashed blue lines) due to residual CFC abnormalities not defined or predicted before revascularization. Non-severe CFC was associated with higher survival probability (*Figure 2* solid black line) that was not changed by revascularization (*Figure 2* dashed black line).

### Coronary flow capacity maps and associated survival probability before and after revascularization to explain and predict low survival probability after revascularization

*Figure 3* displays the range of quantitative regional severity-size of perfusion abnormalities and illustrative CFC ‘rainbow’ map examples in 283 cases from before and after revascularization (*Table 2*). Each size-severity fraction of colour-coded CFC maps contributes cumulatively to different *Observed* CFC-associated survival probabilities for each individual PET as listed beside each image.

**Table 2:** Characteristics of 283 pre- and post-revascularization positron emission tomography pairs

*N* = 283 PET pairs	*N* = 283 pre-procedure		*N* = 283 post procedure		Pre vs. post	
Characteristic	Avg or #	SD or %	Avg or #	SD or %	Test	*P*-value
Age	65.2	9.9	66.7	9.9	*t*-test	.076
BMI	29.0	4.7	28.7	4.7	*t*-test	.484
Male	234	83%	234	83%	chi2	1.000
Hx of PCI	182	64%	261	92%	chi2	*<.00001*
Hx of CABG	94	33%	134	47%	chi2	*.001*
Hx of MI recent	12	4%	12	4%	chi2	1.000
Hx of MI distant	80	28%	109	39%	chi2	*.010*
Hx of hypertension	256	91%	259	92%	chi2	.660
Hx of dyslipidemia	281	99%	281	99%	chi2	1.000
Hx of diabetes	163	58%	167	59%	chi2	.733
Hx of smoking	186	66%	182	64%	chi2	.724
medication_statin	242	86%	264	93%	chi2	*.003*
medication_antiplatelet	252	89.%	278	98%	chi2	*<.00001*
medication_betablocker	190	67%	210	74%	chi2	.065
medication_ACEIorARB	194	69%	193	68%	chi2	.928
medication_ccb	54	19%	54	19%	chi2	1.000
medication_diuretic	99	35%	102	36%	chi2	.792
Stress EF	59%	13%	60%	13%	*t*-test	.358
Abnor relative stress image	246	87%	228	81%	chi2	*.040*
Coronary calcium > 120 HU	276	98%	283	100%	chi2	*.003*
Hx_CAD	230	81%	283	100%	chi2	*.0001*
Hx current angina typical	85	30%	35	12%	chi2	*<.00001*
Hx current angina atypical	23	8%	29	10%	chi2	.383
Hx typical/atypical angina	108	38%	64	23%	chi2	*<.00001*
Hx current dyspnoea	97	34%	70	25%	chi3	*.013*
Hx past dyspnoea	58	21%	69	24%	chi2	.268
PET stress angina #	241	85%	132	47%	chi2	<.00001
PET stress ST Δ #	241	85%	153	54%	chi2	<.00001
Rest relat defect ≤ 60% of LV	6.9%	9.2%	7.1%	9.7%	*t*-test	.792
Strs rel defect ≤ 60% of LV	21.4%	17.2%	14.9%	15.6%	*t*-test	<.00001
CFR minimum quad avg	1.6	0.7	1.8	0.6	*t*-test	*.0001*
CFR minimum quad avg < 2	207	73%	167	59%	chi2	*.0001*
CFR global avg	2.0	0.7	2.1	0.6	*t*-test	*.017*
CFR maximum	3.1	0.97	3.1	0.9	*t*-test	.918
Severe CFC fraction of LV	11.2%	15.9%	6%	12%	*t*-test	*<.00001*
Mild CFC > 15% of LV	226	80%	198	70%	chi2	*.007*
Mod CFC > 15% of LV	73	26%	47	17%	chi2	*.008*
Severe CFC > 0% of LV	184	65%	142	50%	chi2	*.0001*
Strs cc/min/g MQA	1.24	0.57	1.42	0.58	*t*-test	*.0001*
Strs mL/mim/g global avg	1.59	0.57	1.70	0.57	*t*-test	*.014*
Strs mL/min/g maximum	2.48	0.69	2.52	0.68	*t*-test	.469

The italics of <.00001 indicate a highly significant difference in the clinical history of having additional revascularization procedures between the two paired PET scans.

BMI, body mass index; CFR, coronary flow reserve; CFC, coronary flow capacity; HU, Hounsfield units; MI, myocardial infarction; MQA, minimum quadrant average; Rest relat, % of LV with rest relative defect ≤ 60% of maximum activity; Strs rel, % of LV with stress relative defect ≤ 60% of maximum activity; quad avg, quadrant average.

Such favourable outcomes as in *Figure 3A* occurred only after complete revascularization of large regions of severely reduced CFC (blue) with surrounding regions of normal CFC (red), thereby indicating no severe diffuse flow limiting narrowing or multiple stenosis. Commonly, the revascularization only partially improved CFC due to residual abnormalities limiting improvement in survival probability (*Figure 3B–D*). Flow-limiting stenosis superimposed on diffuse CAD improved with residual abnormalities and correspondingly limited improvement in survival probability (*Figure 3E*). Predominant diffuse mildly reduced CFC (yellow) of diffuse CAD commonly did not improve after revascularization, with no change in low survival probability (*Figure 3F*). Stent procedures done despite normal CFC commonly showed worsened CFC and reduced survival probability due to potential risks of procedures including jailed arterial branches that made CFC and survival probability worse (*Figure 3G*).

For 283 paired pre- and post-revascularization PET pairs, the KS plots in *Figure 4* compare CFC size-severity cumulative histograms of PET that were better (*Figure 4A*), unchanged (*Figure 4B*), or worse (*Figure 4C*) after revascularization. The CFC-associated post-revascularization survival probability was improved or worse for baseline pre-revascularization CFC severe (*Figure 4D*), for non-severe CFC (*Figure 4E*), and for baseline normal CFC (*Figure 4F*).

For 184 of 283 (65%) paired PET with CFCsevere in pre-revascularization PET, the CFC histogram significantly improved in post-revascularization PET (*Figure 4A*) (KS = 0.14, *P* < .001) with correspondingly improved average survival probability (*Figure 4D*) (*P* = .00006). For the remaining 99 of 283 (35%) paired PET scans with no severely reduced CFC in pre-revascularization PET, the CFC histogram did not improve (*Figure 4B*) (KS = 0.05, *P* = .14), had unchanged or worsened survival probability (*Figure 4E*) with 40/99 (40%) better and 59/99 (60%) worse (*P* = .00006). For 34 of 283 PET images with normal CFC in ≥90% of the LV (12%), average CFC histogram was significantly worse on post-revascularization PET (*Figure 4C*) (KS = 0.24, *P* < .00001), had worse survival probability (*Figure 4F*) with 7/34 (21%) better and 27/34 (79%) worse.

Of 283 pre-post revascularization PET pairs, only 5.7% with CFC severe normalized CFC with improved survival probability of +Δ0.4 or a 10-year survival probability of ≥0.9 after revascularization. The average improved, no different, or worse changes in the CFC histograms and associated survival probabilities for different severity thresholds were due to different proportions of individual cases with binary better or worse CFC and associated better or worse survival probabilities, reflecting variable effectiveness of revascularization.

Of 283 pre- and post-revascularization PET pairs with severely reduced CFC that improved after revascularization, the survival probability improved by 12% (0.59 ± 0.19 to 0.66 ± 0.22, *P* < .001). However, for pre-revascularization PET with no severely reduced CFC that did not improve on post-revascularization PET, survival probability also showed no improvement (0.83 ± 0.11 to 0.81 ± 0.13, *P* = .14). For all 283 pre- and post-revascularization PET pairs, the net effect was the average of these discordant outcomes, with 160/283 (57%) having better and 123/283 (43%) worse CFC with improved average survival probability from 0.67 ± 0.2 to 0.71 ± 0.2 (*P* < .001). For pre-revascularization PET with normal CFC for ≥90% of LV and survival probability of 0.92 ± 0.01, revascularization was done in a subset of 34/283 patients (12%) associated with worsening CFC and reduced survival probability to 0.85 ± 0.11 (*P* = .00001).

### Microvascular dysfunction, gender, and ethnicity

Severe or moderate transmural CFC abnormalities characterized this population of pre- and post-revascularization PET-pairs. As previously reported, in the absence of regional flow-limiting stenosis or diffuse CAD (no regional severely blue or moderately green reduced CFC), microvascular dysfunction limits coronary blood flow to mildly reduced CFC (yellow).^6,15,16^ This flow limitation due to microvascular disease maintains coronary pressure and thereby maintains normal subendocardial perfusion and relative transmural perfusion gradient. In contrast, diffuse epicardial CAD without focal flow-limiting stenosis decreases hyperaemic coronary pressure and hence subendocardial perfusion quantified as % of LV with reduced relative subendocardial perfusion outside ± 2SD of 125 healthy young volunteers separate from the study participants.^6,15,16^

For diffusely reduced CFC or CFR, reduced relative subendocardial perfusion or transmural perfusion gradient during vasodilatory stress distinguishes diffuse epicardial CAD (reduced subendocardial relative transmural perfusion gradient) from microvascular dysfunction (no relative transmural perfusion gradient).^6,15,16^

For the 283 pre- and post-revascularization PET pairs, the great majority of PET scans had either transmural or subendocardial perfusion abnormalities outside ± 2SD of healthy volunteers indicating flow-limiting stenosis or diffuse CAD. Nine (3%) had microvascular dysfunction limiting perfusion in the absence of moderate or severe stress regional defects or reduced subendocardial perfusion either before or after revascularization (see Supplementary data online, *Table S2*). These findings are consistent with diffuse epicardial CAD and reduced subendocardial perfusion explaining over 95% of no-stenosis angina.^6,15,16^

No gender differences were observed for % of LV with moderately or severely reduced CFC, % of participants with myocardial scar ≥10% of LV, % of participants with microvascular dysfunction, or for survival probability (see Supplementary data online, *Table S2*). Ethnic distribution included Caucasian 70%, Asian 11%, Hispanic 9%, Black 8% and other 2%.

### Coronary bypass surgery vs. percutaneous coronary intervention

Significant improvement was observed in CFC maps (*P* = .001) and *Observed* CFC-associated survival probability (*P* < .00001) from before to after coronary artery bypass surgery (*P* < .03) but not comparably after PCI (*P* = .3) (see Supplementary data online, *Figure S1*).

### Observed coronary flow capacity-associated survival probability after actual revascularization vs. *Virtual* survival probability after hypothetical revascularization in 283 pre- and post-revascularization positron emission tomography pairs

For the 283 pre- and post-revascularization PET pairs, the *Observed* survival probability correlated with CFC severity before and after actual revascularization that was worse with myocardial scar (*Figure 5*). The *Observed* CFC-associated survival probability after actual revascularization was compared to the *Optimal and Realistic Virtual* survival probabilities after hypothetical complete or incomplete revascularization or residual diffuse CAD, respectively (*Figure 6*). The *Optimal Virtual* survival probability averaged 0.81 ± 0.88, i.e. significantly higher than 0.71 ± 0.21 (*P* < .001) of the *Observed* CFC-associated survival probability after actual revascularization due to residual diffuse or multi-stenosis CAD or incomplete revascularization (*Figure 6*) as illustrated by the residual CFC abnormalities in the individual PET pairs of *Figure 3* and KS plots of *Figure 4*. The *Realistic Virtual* survival probability of 0.74 ± 0.15 approximated the 0.71 ± 0.21 of the *Observed* survival probability after actual revascularization. Bland-Altman plot showed a small mean bias of +0.03 for the *Realistic* over *Observed* survival probability (*P* = .001) due to residual CAD (see Supplementary data online, *Figure S2*).

### Post-revascularization coronary flow capacity in 283 positron emission tomography pairs compared with 2552 post-revascularization positron emission tomography

The average post-revascularization CFC in the 283 PET pairs (*Figure 7*, dash-dot lines) was comparable by KS test to the post-revascularization CFC of the 2552 PET images from patients with prior revascularization (*Figure 7*, solid lines). Both had CFC that was significantly more severe than with no prior revascularization (*Figure 7*, light dashed lines) (*P* < .00001). Both were much worse than 125 healthy young volunteers without risk factors as a reference (*Figure 7*, heavy dashed line with solid squares).

### Repeat revascularization and *Observed* survival probability

For the 2552 patients with prior revascularization, multi-variable Cox regression modelling shows CFC severe associated with severely reduced survival probability (*Figure 8*, solid blue line) compared to no severely reduced CFC (*Figure 8*, black line). Additional revascularization is associated with improved survival probability (*Figure 8*, dashed blue line). However, after revascularization, substantial mortality risk remains due to residual CFC reflecting residual diffuse or segmental disease or incomplete revascularization.

## Discussion

The data confirm our hypothesis. Severely reduced CFC associates with or predicts low 10-year survival probability that is significantly improved after revascularization compared to no revascularization for comparable CFC severity. However, effects of revascularization on CFC and survival probability are heterogeneous with residual CFC abnormalities and associated limited survival due to residual diffuse CAD, multiple stenosis, incomplete or failed revascularization procedures, or disease progression. Only 5.7% of post-revascularization PETs normalize the CFC map. Survival probability is not improved or worse after revascularization in non-severe CFC (*Structured Graphical Abstract*).

In 283 pre- and post-revascularization PET pairs, the change in CFC maps confirms this heterogeneous response with significant residual CFC abnormalities by KS tests comparing CFC severity histogram plots. The associated *Observed* CFC-associated survival probability as a fraction of 1.0 was on average better (higher) after than before revascularization in the same subject. However, individual changes in the pre- and post-revascularization CFC pair and associated changes in survival probability were heterogeneously better or worse after revascularization. Gender, ethnicity, or microvascular dysfunction played no role in these heterogeneous outcomes likely related to the comprehensive CFC maps that integrate and account for cumulative risk factors, stenosis, and diffuse CAD.

Finally, in 2552 post-revascularization PET, substantial residual CFC abnormalities were common in association with *Observed* CFC-associated lower survival probability. Both were significantly improved after repeat revascularization compared with no-repeat revascularization for comparable CFC severity.

Importantly, for pre-revascularization PET with primarily normal CFC and good survival probability, revascularization is associated with worsening CFC and significantly reduced survival probability due to stent-jailed branches, procedure failures, or complications of procedures performed despite normal or adequate CFC.

### Implications for randomized trials

Our data suggest that survival probability might not have improved in reported revascularization trials for chronic coronary syndromes due to several quantifiable pathophysiologic reasons. Patient selection by angiogram without quantitative perfusion may not select sufficient physiologic severity associated with sufficient mortality to be reduced by revascularization comparably to severely reduced CFC by PET. Second, residual diffuse or multi-stenosis CAD and incomplete or failed revascularization may cause substantial remaining severely reduced CFC incurring high residual mortality risk that is reduced by repeat revascularization that can be *virtually* predicted before intervention. These results parallel and offer a mechanistic explanation for only 43% and 58% of revascularized patients achieving anatomic or functional complete revascularization respectively reported for the ISCHEMIA trial.^17^

### Coronary flow capacity as gatekeeper to interventions

Of the large referral cohort of 6979 patients, 77% had known or suspected CAD, coronary calcium, high-risk factors, or non-severe quantitative PET abnormalities. However, only 21% had severely reduced CFC and only 12% had revascularization based on clinical judgement, comorbidities, size, and distal vs. proximal severe stress defects. Moreover, of 283 pre- and post-revascularization paired PETs, only 5.7% completely normalized the CFC after revascularization with associated normalized survival probability. Adding up to 23 additional clinical covariates did not change the mortality benefit after revascularization (see Supplementary data online, *Table S1*). Therefore, the cumulative data suggest that CFC is the final common cumulative physiologic expression of risk factors and CAD severity for predicting mortality risk that is modifiable by revascularization.

### Contributions of coronary pathophysiology to clinical decision making in chronic CAD

This study presents a comprehensive extension of the established concepts of experimental pathophysiology of coronary stenosis,^18^ quantitative angiographic anatomic-pressure-flow relations,^18–23^ fractional flow reserve (FFR),^24^ anatomic simulation of relative CFR (FFR_PET_),^19–23^ subendocardial perfusion,^6,15,16,23^ and severity of diffuse or focal CAD with associated individual survival probability before and after actual and virtual hypothetical revascularization by quantitative CFC^4–12^ for clinical decision making in chronic coronary syndromes.

As the next step in this evolution, the randomized CENTURY trial of a personalized, comprehensive, integrated strategy of lifestyle and medical treatment to goals with interventions for only severely reduced CFC documents significant survival benefit, reduced MI, reduced revascularizations, and angina relief in chronic CAD vs. standard community care (NCT00756379, ClinicalTrials.gov).^25^ The considered ESC Guidelines emphasize functional testing before revascularization, its limited survival benefit, and completeness of revascularization.^26,27^ The current study and the survival benefit of the randomized CENTURY trial^25^ suggest that CFC combining stress mL/min/g and CFR per regional LV pixel provide a precise metric of coronary artery-specific size-severity pathophysiology for optimal medical-lifestyle management with PET-guided interventions reserved for severe perfusion abnormalities at high mortality risk that is reduced by revascularization or for refractory angina.

### Limitations of the study

This report on physiologic CAD severity quantified by CFC is a natural history study of survival probability at a tertiary care referral centre in a large cohort unrestricted by assumed CAD severity thresholds required by randomization, with unknown bias for PET imaging intervention or medical treatment, paralleling potential selection bias for randomized trials. Quantitative invasive or non-invasive physiologic metrics by PET are not widely used, employ diverse protocols, methodology, and criteria for intervention. The study has the limitations of a single tertiary care cardiovascular centre with high prevalence of CAD and quantitative PET perfusion imaging with highly developed, validated analytical software that is not widely available.

The results are conceptually relevant to all invasive and non-invasive physiologic measurement technologies—quantitative magnetic resonance imaging, CT flow, attenuation corrected single photon emission CT, echo flow, invasive Doppler or thermodilution or FFR, and of course quantitative PET used as the gold standard to prove the clinical concepts. However, implementation requires accurate myocardial perfusion in mL/min/g for quantifying CFC per regional pixel that currently only PET is provided.

On the other hand, the data have unique strengths that include a precision of ±10% for quantitative perfusion, all cases undergoing the same protocol, on the same scanner, carried out by the same physicians, technical team, using the same software, relational database, and same prospective systematic follow-up with entry-signed consent over 14-year follow-up.

## Conclusions

Severely reduced baseline CFC as a comprehensive integrated physiologic measure of CAD severity and associated *observed* low survival probability is significantly but heterogeneously improved after revascularization compared to no revascularization for comparable severity. For non-severe CFC, survival probability was high with revascularization having no added survival benefit or causing harm. CFC derived *Virtual* survival probability after conceptual hypothetical revascularization was higher on average than CFC-associated *Observed* survival probability after actual revascularization due to residual CAD or failed revascularization. Substantial residual severe CFC abnormalities were common after revascularization with associated reduced survival probability both of which were significantly but heterogeneously improved after *repeat* revascularization compared with no repeat revascularization for comparable CFC severity.

## Supplementary Data

Supplementary data are available at *European Heart Journal* online.

## Supplementary Material

ehad579_Supplementary_Data

## Data Availability

If accepted for publication, de-identified data and statistical analysis will be made available on request.
