# Supplementary material for: Coronary flow capacity and survival prediction after revascularization: physiological basis and clinical implications
Source: Eur Heart J. 2023 Aug 27;45(3):181–94. doi: 10.1093/eurheartj/ehad579 (PMC10787661; doi:10.1093/eurheartj/ehad579)

***Predicted* and *observed* survival probability based on coronary flow capacity with and without *virtual* *ideal* and *actual* revascularization:**

***Physiologic basis of improvement in survival probability after revascularization in individual patients***

By Gould et al.

Supplementary Files

Supplement Table S1. Severely reduced CFC and mortality benefit with and without revascularization after PET versus no revascularization for comparable CFC severity by multivariable Cox regression modeling with multiple time-dependent covariates.

| ***CFCsevere**** | **HR for revasc vs no revasc** | **P for hazard ratio** | **Confidence Intervals** | |
| --- | --- | --- | --- | --- |
| **Covariates 4** | **0.472** | **0.0009** | **0.302** | **0.737** |
| **Covariates 16** | **0.512** | **0.0038** | **0.325** | **0.806** |
| **Covariates 23** | **0.536** | **0.0067** | **0.341** | **0.841** |

HR = hazard ratio for probability of death for revascularization vs no revascularization

revasc = revascularization

*CFC severe = any pixel with CFR ≤1.27 + stress flow cc/min/g ≤0.83 per pixel as % of LV.

Covariates 4: pcicabg90, age, gender, minimum quadrant average cfr.

Covariate 16: pcicabg90, age, gender, m_severe, BMI, history of smoking, dyslipidemia,

diabetes, prior PCI, CABG, MI within 3 months prior to PET, MI prior to 3 months

before PET, documented CAD by angiogram, typical angina, atypical chest pain,

coronary calcium on CT ≥ 120 Hounsfield units.

Covariates 23: Same covariates as in Covariates 16 plus a covariate each for a statin,

antiplatelet agent, betablocker, ACEI, ARB, calcium channel blocker or diuretic.

Supplement Table S2. Gender and microvacular dysfunction of PETs in 234 males and 49 frmales before and afer revacularization.

|  | Male (n = 234) | | | Female (N = 49) | |  |
| --- | --- | --- | --- | --- | --- | --- |
| Characteristic | Before revasc | After revasc | P for ∆  B vs A | Before revasc | After revasc | P for ∆  B vs A |
| CFC mild % of LV | 34±20% | 33±20% | P = 0.47 | 31±20% | 29±20% | P = 0.54 |
| CFC sev+mod % LV | 21±20% | 14±20% | ***P<0.001*** | 20±22% | 13±20% | ***P = 0.004*** |
| #, % w scar>10% LV | 20 (8%) | 27 (11%) | P = 0.28 | 4 (8%) | 6(12%) | small # |
| % w microvasc dys* | 8 (3%) | 4 (3%) | small # | 1 (2%) | 1 (2%) | small # |
| survival probability | 0.67±0.2 | 0.71±0.2 | P=0.001 | 0.69±0.2 | 0.72±0.2 | P = 0.18 |
|  | | | | M vs F  P = 0.58 | M vs F  P = 0.76 |  |

B = before ervascularization. A = afer revascularization.

M = male. F = female.

*% of PETs with primarily microvascular dysfunction defined as CFCmild with normal subendocardial perfusion and no moderate to severe transmural stress defects due to flow limiting senosis or diffuse CAD and no reduced hyperemic subendocardial perfusion due to diffuse epicardial CAD.

Supplementary Figure S1. Comparison of CFC size-severity histogram distributions by the Kolmogorov – Smirnov test (A) and survival probability box plots (B) before and after coronary artery bypass surgery (CABG) and percutaneous coronary intervention (PCI).


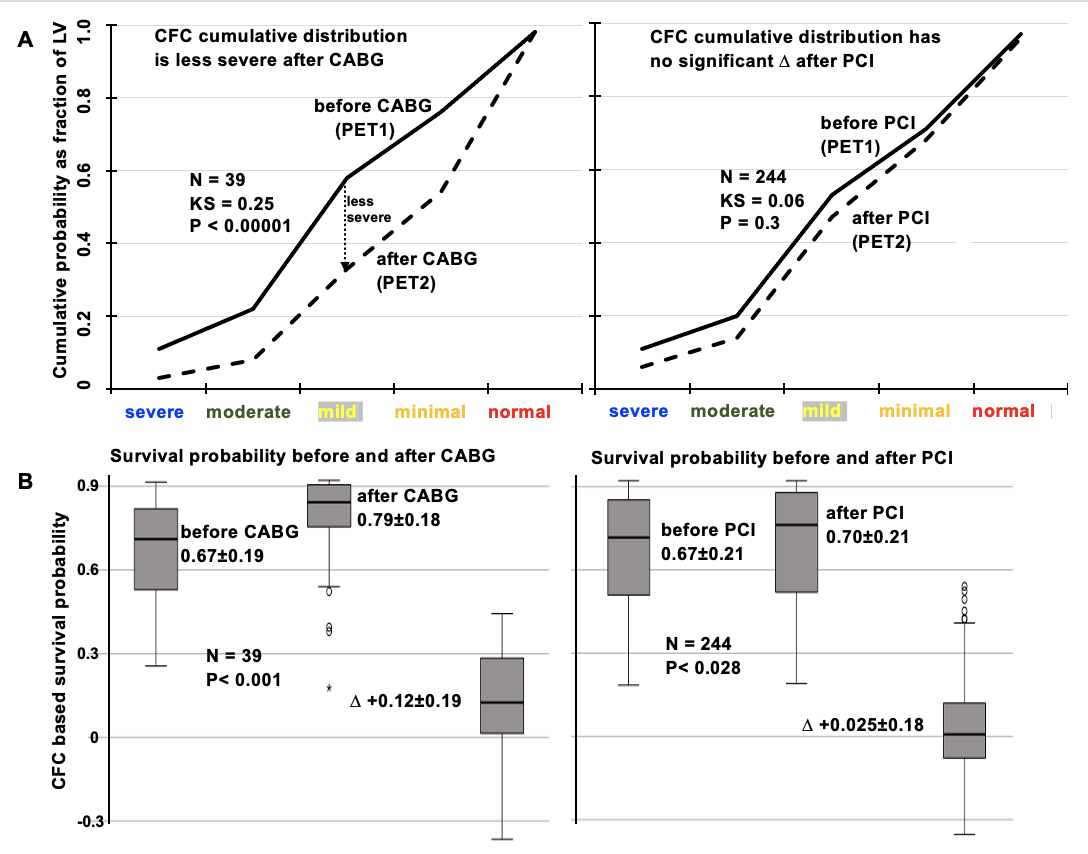


Supplementary Figure S2

*The Realistic Virtual* survival probability after hypothetical revascularization accounting for diffuse CAD and potential incomplete revascularizaton was determined in the multivariable Cox egression model by replacing the % of severely reduced CFC pixels in pre-revascularization CFC maps with a proportionate distribution of normal to mild CFC pixels surrounding the severe CFC abnormality. On the Bland Altman plot, the *Realistic* V*irtual* survival probability after hypothetical revascularization is slightly higher than the CFC-associated *Observed* survival probability after actual revasculariz with a small bias of +0.03 (P=0.001) due to residual CAD or incomplete revascularization not completetly accounted for in the *Realistic Virtual* Cox model.


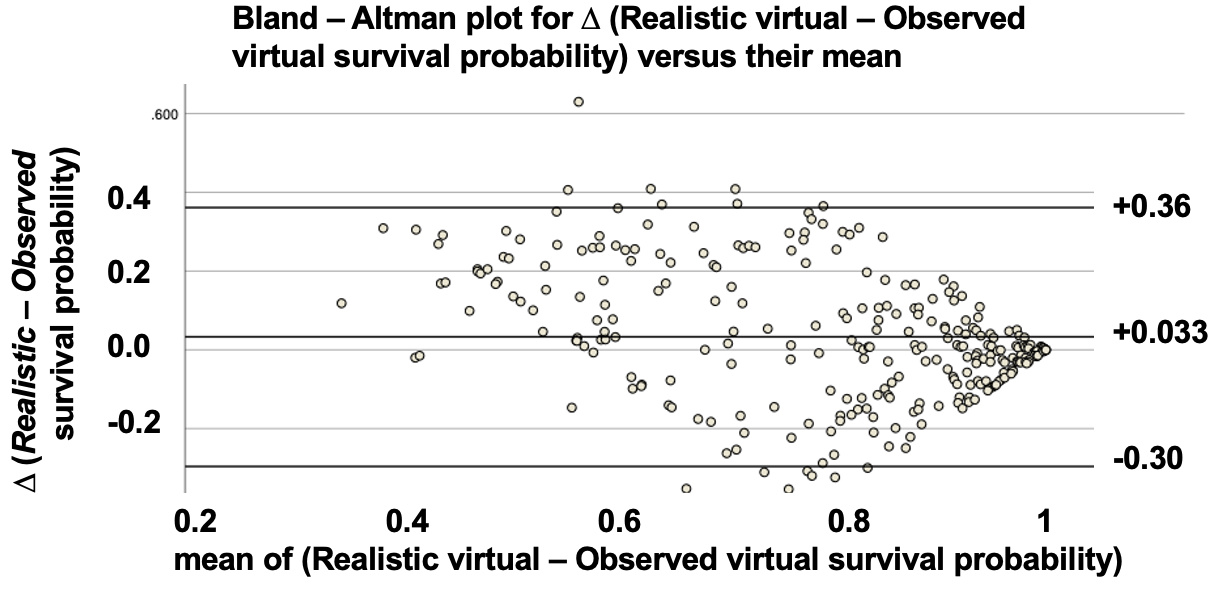

Supplement: ehad579_Supplementary_Data [file ehad579_supplementary_data.docx]
